# Determination of Seven Antidepressants in Pericardial Fluid by Means of Dispersive Liquid–Liquid Microextraction and Gas Chromatography–Mass Spectrometry

**DOI:** 10.1093/jat/bkab003

**Published:** 2021-01-07

**Authors:** P Cabarcos-Fernández, M J Tabernero-Duque, I Álvarez-Freire, A M Bermejo-Barrera

**Affiliations:** Forensic Toxicology Service, Institute of Forensic Sciences, Faculty of Medicine, University of Santiago de Compostela, C/San Francisco s/n, Santiago de Compostela, A Coruña 15782, Spain; Forensic Toxicology Service, Institute of Forensic Sciences, Faculty of Medicine, University of Santiago de Compostela, C/San Francisco s/n, Santiago de Compostela, A Coruña 15782, Spain; Forensic Toxicology Service, Institute of Forensic Sciences, Faculty of Medicine, University of Santiago de Compostela, C/San Francisco s/n, Santiago de Compostela, A Coruña 15782, Spain; Forensic Toxicology Service, Institute of Forensic Sciences, Faculty of Medicine, University of Santiago de Compostela, C/San Francisco s/n, Santiago de Compostela, A Coruña 15782, Spain

## Abstract

Although blood is often used to detect and quantify the presence of drugs, there are some instances where samples obtained from other biological matrices, like pericardial fluid (PF), are necessary since adequate blood samples may not be available. PF is an epicardial transudate, which contains plasma components that include toxicological substances making this sample useful when blood samples are not available. This fluid is a well-preserved postmortem sample and can easily be collected in larger amounts without significant contamination, compared with other body fluids. Although studies involving PF began around the 1980s, the adequacy of such fluid as a biological matrix has been poorly investigated. Antidepressants are frequently detected in postmortem samples from forensic cases. Nowadays, they constitute some of the most commonly prescribed drugs worldwide. A total of seven antidepressants (venlafaxine, mirtazapine, olanzapine, paroxetine, sertraline, fluoxetine and citalopram) were evaluated in this study. A new extraction method involving dispersive liquid–liquid microextraction (DLLME) is presented in which chloroform and acetonitrile are determined to be the best extraction and dispersing solvents. The experimental design was achieved using StatGraphics 18. The response surface methodology enabled us to know the optimal volume for the two solvents used in the DLLME. The detection technique used was gas chromatography**–**mass spectrometry with electron impact ionization as ionization source. A temperature gradient has been used and the total chromatographic separation time was 19.43 min. Validation results met the international validation guidance (Food and Drug Administration (FDA)). Under the optimal condition, the method offered good validation parameters showing a new efficient, simple, rapid and sensitive method. The analytical method was applied to 31 PF samples. Twenty-one samples were positive with concentrations between 0.19 and 8.48 µg/mL. Venlafaxine and olanzapine were the antidepressants most frequently found.

## Introduction

Depression constitutes nowadays one of the most common mental disorders either as a major condition or as a neuropsychiatric symptom characteristic of several diseases. In fact, major depressive disorder is a chronic, prevalent and pervasive brain-based disorder that significantly incapacitates the person affected (1). It is estimated that more than 350 million people worldwide are currently diagnosed with such condition, being women more frequently affected than men (2). At its worst, depression may lead to suicide. Close to 800,000 people commit suicide each year, being the second leading cause of death in 15–29-year-olds (3).

Tricyclic antidepressants (TCAs) were discovered in the 1950s, but their significant side effects led to a sustained effort in search for more selective drugs. This leads to the discovery of serotonin reuptake inhibitors, some of which (fluoxetine, sertraline and citalopram) are very commonly prescribed as first-choice drugs to treat depression. In addition, newer antidepressants, such as mirtazapine and venlafaxine, do affect both the serotonin and norepinephrine systems within the central nervous system, without the associated anticholinergic and cardiovascular side effects of older drugs such as TCAs. Mirtazapine has a dual mode of action. It is noradrenergic and specific serotonergic antidepressant that acts by antagonizing the adrenergic α2-autoreceptors and α2-heteroreceptors as well as by blocking 5-HT_2_ and 5-HT_3_ receptors. It enhances, therefore, the release of norepinephrine and 5-HT_1A_-mediated serotonergic transmission (4). Venlafaxine is a phenylethylamine derivative. It is a serotonin norepinephrine re-uptake inhibitor. Venlafaxine was approved by the FDA (Food and Drug Administration) for the following conditions: major depression, generalized anxiety disorder, panic disorder and social phobia (5). Olanzapine is a second-generation antipsychotic neuroleptic or “atypical antipsychotic”. These medications have more receptor-binding targets than first-generation treatments (e.g., haloperidol) do and have complex pharmacologic mechanisms of action. Olanzapine has the most binding targets in its class, binding to 10 serotonin receptors, 4 dopamine receptors, 4 acetylcholine receptors, 4 α-adrenergic receptors, 1 histamine receptor and 2 different neurotransmitter transport receptors (6).

Within the forensic practice, it is important to investigate postmortem drug concentrations to discriminate whether the cause of death was due to intoxication, side effects of the undergoing treatment or lack of compliance. Urine and blood specimens are the main biological samples used in forensic autopsy cases, being peripheral blood the most used in postmortem research. Moreover, special care must be taken in the interpretation of the data when using postmortem blood samples as these concentrations may change due to several factors such as chemical/enzymatic degradation of substances, redistribution phenomena and postmortem diffusion from solid organs or gastric content, especially with cardiac blood since it is more affected by postmortem redistribution (7). However, it should be borne in mind that blood analysis will be always a fundamental and essential step since it enables one to know the toxicological status of the deceased at the time of death. In addition to blood or urine samples, one may consider pericardial fluid (PF) as a useful alternative matrix in cases where adequate peripheral blood samples cannot be obtained. The potential use of PF samples has been considered within relatively few reports (7–13). Previous studies showed good correlation with the drug concentrations in peripheral blood (8, 10, 14, 15). PF is a well-preserved postmortem material in cases without structural damage due to injury or medical intervention and can easily be collected in large amounts without significant contamination as it is contained within a tight compartment (pericardial sac), compared with other body fluids (13). With a volume of 15–35 mL, it is believed to be a transudate generated by the net result of the hydrostatic pressure and the osmotic gradient between plasma and PF. The composition of the normal human PF is difficult to define. The hematological and biochemical analyses of the PF show that the concentration of electrolytes and small molecules (urea, uric acid, glucose and creatinine) corresponds to an ultrafiltrate of plasma (7, 13).

Previous works using liquid–liquid extraction or solid phase extraction have been reported (7–9, 12). In particular, efforts were oriented towards the development of efficient and miniaturized sample preparation methods. Liquid-phase microextraction (LPME) was introduced according to this perspective. Dispersive liquid–liquid microextraction (DLLME) is one of the LPME methods and has attracted recent interest within the analytical chemists’ community (16). The method was introduced by Assadi and co-workers in 2006 (17). It is a simple and fast microextraction technique based upon the use of an appropriate extractant (an organic solvent with high density) and a disperser solvent with high miscibility in both extractant and aqueous phases. DLLME consists of two steps: (i) injection of an appropriate mixture of extracting and disperser solvents into an aqueous sample, containing the analytes. In this step, the extracting solvent is dispersed into the aqueous sample as very fine droplets and the analytes are enriched into it. After the formation of a cloudy solution, the surface area between the extracting solvent and the aqueous sample becomes very large, so the equilibrium state is quickly attained and, therefore, the extraction time is very short. In fact, this is the principal advantage of this method. (ii) Centrifugation of the cloudy solution, thereby obtaining a settled phase that is deposited at the bottom of a conical tube. Other advantages include simplicity of operation, rapidity, low cost, high recovery, high enrichment factor and environment benignity (18). Like the other analytical methods, DLLME has some disadvantages, which result from the requirements related to the organic extraction and disperser solvents. The extraction solvent used should have some special requirements, such as a density larger than water for simple separation of the extraction phase after centrifugation and to form a cloudy solution in the presence of the disperser solvent. The potential organic solvents meeting this requirement are limited since they are hazardous chemicals (such as halogenated hydrocarbons). It is precisely because of this the choice of the extraction solvent becomes the method’s primary limitation (16). According to detection techniques, several methods were used involving liquid chromatography as well as gas chromatography (7–9, 12).

The aim of this study was to develop a gas chromatography–mass spectrometry (GC–MS) method for the determination and quantification of antidepressants in PF using DLLME as a new extraction method. The validation of the method was carried out according to the guidelines of the FDA (19). The method was then applied to PF samples collected from deceased people.

## Material and Methods

### Chemicals

Acetonitrile and chloroform gradient grade solvents were purchased from Merck^®^ (Madrid, Spain) and sodium chloride from Panreac^®^ (Barcelona, Spain). Fluoxetine, venlafaxine, mirtazapine, sertraline, citalopram, olanzapine, paroxetine and proadifen (SKF), used as internal standard, were obtained from Cerilliant^®^. Distilled water was processed through a Milli-Q water system (Millipore, Bedford, MA, USA).

### Matrix collection

To carry out the validation procedure, antidepressant-free PF obtained from autopsies was used. For this purpose, a mixture of PF from deceased subjects was collected and stored at –20°C until use, following a previous peripheral blood and urine analysis that was negative to antidepressants.

### Sample preparation

PF used for validation was previously ultracentrifuged for 5 min at 14,000 rpm. Aliquots of 0.3 mL were used for analysis, collected in a glass tube and spiked with Proadifen (SKF) (20 µL Sol. 10 µg/mL). Then, it was diluted with water (1.1 mL) and 150 mg of NaCl was also added to facilitate analytes’ transition from aqueous phase to organic phase. This mixture was submited to the DLLME. After an experimental design using StatGraphics (StatGraphics Technologies, Inc.), the optimal conditions were as follows: 175 uL of chloroform and 750 uL of acetonitrile as the best extracting and disperser solvents, respectively. Both solvents were rapidly injected into the sample with a Pasteur pipette and the mixture was gently shaken. Then, the mixture was centrifuged, and the droplet formed was collected by a 100 µL syringe and transferred to an 8 mL glass tube. The organic solvent was evaporated under a stream of nitrogen in a heated aluminum block at 40°C (VLM GmbH, HP series). The dried residue was redissolved with 40 µL of methanol prior to injection of a 2 µL aliquot into the GC–MS system.

### Instrumentation

Chromatographic analyses were performed using an electron impact ionization gas chromatograph model 7890 B from Agilent Technologies^®^ (Las Rozas, Spain) interfaced to a mass selective detector (MSD) model 5977 B, also from Agilent Technologies^®^. An HP5-MS capillary column (30 m × 250 µm i.d., 0.5 µm film thickness; Agilent Technologies^®^) with helium as carrier gas (1 mL/min) was used for the gas chromatographic separation. The injector temperature was set at 250°C and a purge time of 2 min was used. Samples were injected in the splitless mode. The following temperature program was applied: the initial temperature of the column was 100°C for 1 min, then ramped-up progressively at 35°C/min up to 220°C, held constant for 1 min, and then ramped-up again at 8°C/min up to 260°C and held for 2 min. Finally, the temperature was ramped-up at 5°C/min up to 280°C for 3 min. After that, the temperature was increased to 290°C for 5 min to clean the column. The total chromatographic separation time was 19.43 min, and the total run time was 24.43 min. The MSD was kept at 300°C, the ion source at 230°C and the quadrupole at 150°C.

### Identification of compounds

**Figure 1.**
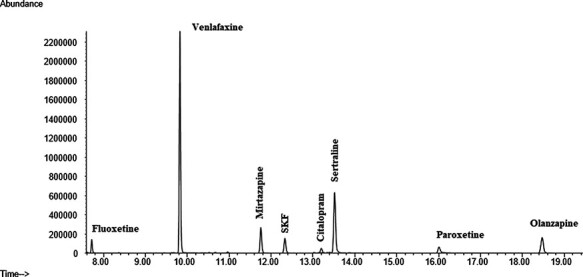
Extracted ionic chromatogram of all analytes.

Initially, neat standards of all antidepressants were injected and analyzed using the full scan mode of the GC–MS, which scanned from 50 to 550 amu. Quantifier and qualifier ions used for each analyte were selected based on their abundances and mass-to-charge ratios (*m*/*z*). Because of their reproducibilities and lack of interference, high mass ions were selected whenever possible. The ions selected for each compound studied and the retention times are shown in Table I. Upon selection of ion, the mass spectrometry was run in selected ion monitoring mode (Figure 1).

**Table I.** Retention Times and Ions Selected for Monitorization

Analyte	Quantifier ion, *m*/*z*	Qualifier ions, *m*/*z*	Retention time, min
Fluoxetine	104	148, 309	7.78
Venlafaxine	58	134, 179	9.97
Mirtazapine	195	208, 245	11.78
SKF	86	99, 165	12.30
Sertraline	274	276, 262	13.55
Citalopram	58	238, 324	13.20
Paroxetine	192	138, 329	16.09
Olanzapine	242	229, 207	18.50

## Results

### Experimental design

**Figure 2.**
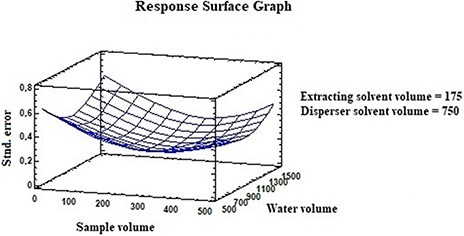
Response surface graph.

To find the best conditions for DLLME, 18 replicates were performed using a 2 × 4 factorial design with four factors: sample volume, water volume, extracting solvent volume and disperser solvent volume. The experimental design was achieved using Statgraphics 18. To make this design rotatable, for each factor, two axial points were chosen, and the runs were randomized to exclude the block effects. Response surface methodology (RSM) is a combination of mathematical and statistical techniques used to study the relationship between two or more responses that depend on several factors or independent variables. The final goal of RSM is to optimize responses determining the best conditions in the operation of the system. Our design of response surface was obtained using the statistical software StatGraphics (Figure 2).

### Validation of the method

Validation was achieved according to the FDA Guideline for Bioanalytical Method Validation (19). The suitability of the method for quantitative analysis was studied by testing selectivity, linearity and sensitivity, precision and accuracy and recovery.

#### Selectivity

**Figure 3.**
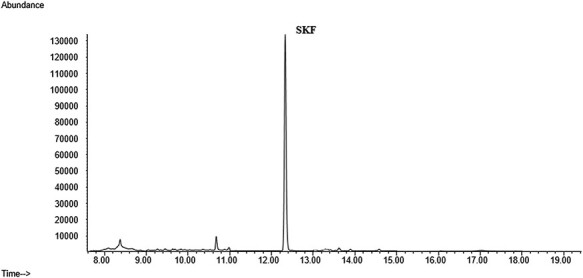
Chromatogram of a blank PF.

Selectivity is the ability of an analytical method to differentiate and quantify the analyte in the presence of other components in the sample. It should confirm that the assay is free of potential interfering substances, include endogenous matrix components, metabolites, decomposition products and medication and other exogenous xenobiotics. The selectivity of the method was demonstrated by analyzing six blank PF samples (19). No interfering peaks were found at the retention time for all analytes (Figure 3).

#### Linearity

Standard addition curves were obtained in six runs with the described method using drug-free control PF spiked with standard solutions to obtain the range of concentrations shown in Table II. The curves were obtained by fitting the ratio of the peak areas of analytes to that of IS versus concentrations. A linear response was observed in the studied range with a good correlation coefficient (higher than 0.99 in all cases). The sensitivity of the method was determined by calculation of the limit of detection (LOD) and the lower limit of quantification (LLOQ). LOD was determined by an empirical method that consists of analyzing a series of PF samples containing decreasing amounts of the analytes. LOD was the lowest concentration that presented a signal-to-noise ratio higher than 3 for at least three diagnostic ions for each substance. The LLOQ was the lowest concentration of analytes in a sample that can be determined with appropriate precision (20%) and accuracy (80–120%) (19) (Table II).

**Table II.** LOD, Limit of Quantification and Calibration Results for the Antidepressants Studied

Analyte	LOD (µg/mL)	LLOQ (µg/mL)	C. coef.	Range of calibration (µg/mL)
Fluoxetine	0.05	0.2	0.991	0.2–10
Venlafaxine	0.01	0.02	0.991	0.02–10
Mirtazapine	0.01	0.02	0.997	0.02–5
Sertraline	0.005	0.2	0.992	0.2–5
Citalopram	0.01	0.04	0.995	0.04–5
Olanzapine	0.02	0.2	0.993	0.2–10
Paroxetine	0.2	1	0.997	1–10

C. coef., correlation coefficient.

#### Precision and accuracy

The accuracy of an analytical method describes the closeness of mean test results obtained by the method to the true value (concentration) of the analyte. The precision of an analytical method describes the closeness of individual measures of an analyte when the procedure is applied repeatedly to multiple aliquots of a single homogeneous volume of biological matrix. Precision and accuracy were determined by inter- and intra-day assay. Inter-day precision and accuracy were calculated by analyzing negative PF samples spiked with the antidepressants at three concentrations; the LLOQ, the upper limit of quantification and an intermediate level were assessed by analyzing five replicates each day in five different days for each level of concentration. Intra-day precision and accuracy were determined at three concentrations, by preparing and analyzing five replicates for each level on the same day. Precision, expressed as the coefficient of variation of the measured values, was expected to be less than 15% at all concentrations, except for the LLOQ for which 20% was acceptable. In the same way, accuracy was evaluated using the mean relative error (ME), which had to be less than 15% of the theoretical values at each concentration level except for the LLOQ, for which 20% was acceptable (19). Data presented in Table III (Supplementary Material) satisfied the international validation rules.

**Table III.** Intra- and Inter-Day Precision and Accuracy (ME) of the Method

	Intra-day study (*n* = 5)		Inter-day study (*n* = 5)	
Concentrations (μg/mL)	ME (%)	RSD (%)	ME (%)	RSD (%)
Fluoxetine				
0.2	17	5	14	10
5	6	5	8	4
10	6	7	1	5
Venlafaxine				
0.02	18	8	14	7
5	6	3	5	3
10	2	5	1	4
Mirtazapine				
0.02	19	3	16	6
2.5	11	2	6	2
5	1	4	3	6
Sertraline				
0.2	17	7	18	7
2.5	8	5	6	3
5	2	8	1	4
Citalopram				
0.04	17	10	13	7
2.5	5	4	4	3
5	1	4	2	3
Paroxetine				
1	13	12	13	12
5	8	15	9	12
10	14	12	1	7
Olanzapine				
0.2	12	14	19	5
5	14	3	9	6
10	6	3	2	7

RSD: Relative Standard Deviation.

#### Recovery

The recovery of an analyte is the detector response obtained from an amount of the analyte added to an extracted from the biological matrix, compared to the detector response obtained for the true concentration of the pure authentic standard (19). The recovery of the method was examined by comparing the analytical results for extracted samples at three levels of concentration (high, medium and low) five times within three days with theoretical concentration that represent 100% recovery. The data obtained demonstrates that the extraction procedure is particularly efficient, providing a recovery values ranged from 85 to 105% for all compounds. The results are shown in Table IV (Supplementary Material).

**Table IV.** Intra and Inter-Day Accuracy (Analytical Recovery) of the Method

	Intra-day study (*n* = 5)	Inter-day study (*n *= 5)
Concentrations (μg/mL)	Recovery (%)	Recovery (%)
Fluoxetine		
0.2	98	89
5	93	91
10	94	100
Venlafaxine		
0.02	92	95
5	93	95
10	98	99
Mirtazapine		
0.02	85	94
2.5	89	94
5	92	97
Sertraline		
0.2	97	98
2.5	91	94
5	98	99
Citalopram		
0.04	93	105
2.5	95	96
5	99	98
Paroxetine		
1	93	93
5	91	91
10	94	101
Olanzapine		
0.2	89	89
5	85	90
10	87	98

### Application to real cases

**Figure 4.**
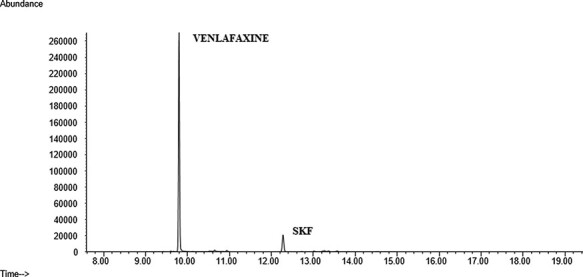
Chromatogram of a real sample (positive for venlafaxine).

**Figure 5.**
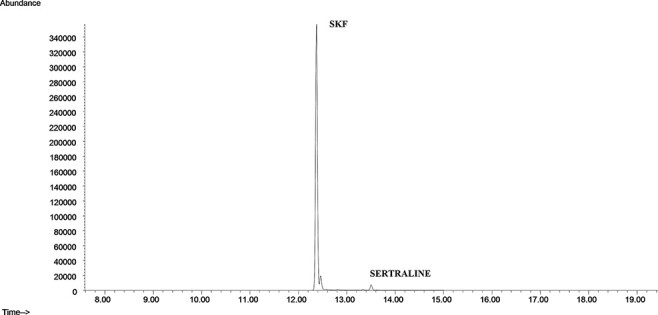
Chromatogram of a real sample (positive for sertraline).

**Figure 6.**
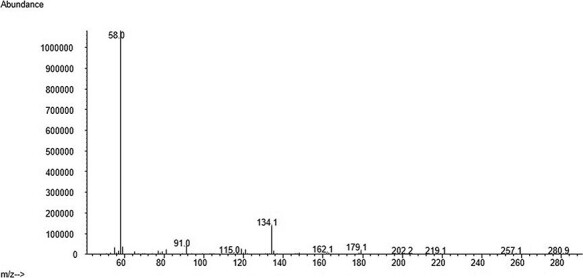
Venlafaxine mass spectra.

**Figure 7.**
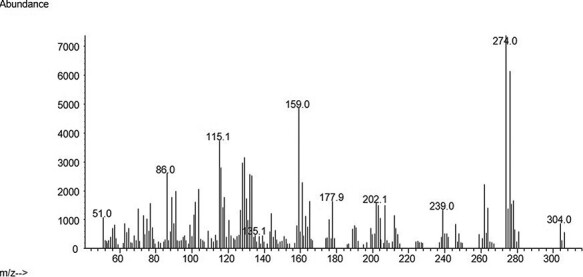
Sertraline mass spectra.

The developed method was used to analyze 31 real PF samples obtained from the Forensic Toxicology Service of the Institute of Forensic Sciences of Santiago de Compostela from cases including suicide, overdose or death from unknown causes. Some information is described in Table V. From the 31 cases, 21 were positive for at least one of the substances analyzed. Venlafaxine and olanzapine were the two antidepressants most frequently found in 12 and 4 cases, respectively. There were also positive cases for mirtazapine, fluoxetine, sertraline and citalopram. Three samples were positive for several compounds at the same time. Figures 4 and 5 show real cases analyzed in our laboratory with their corresponding mass spectra (Figures 6 and 7). One of them corresponds to a female whose cause of death was hanging after having consumed high amounts of several drugs and the other case belongs to a male poisoned by carbon monoxide.

**Table V.** Real Cases

Case no.	Age	Sex	Sample	Cause of death	General information and treatment	Substance detected
	(years)					[µg/mL]
1	82	Female	Blood, urine and PF	Pulmonary embolism	*Depressive disorder*	Mirtazapine [0.48]
2	83	Female	Blood, urine and PF	Traumatic brain injury	Duloxetine, bromazepam, quetiapine, tramadol and metamizol	–
3	42	Female	Blood, urine and PF	Suicide	*Psychiatric treatment for schizophrenia*. Lormetazepam and paliperidone	Venlafaxine [0.27]
4	32	Female	Blood and PF	Suicide	*Depressive disorder*. Risperidone, benzodiazepines and olanzapine	Olanzapine <LLOQ
5	56	Female	Blood, urine and PF	Suicide	Venlafaxine and benzodiazepines	Venlafaxine [3.44]
6	45	Male	Blood, urine, vitreous humor and PF	Suicide	*Past history of drug abuse*	Venlafaxine [0.27]
7	52	Male	Blood, vitreous humor and PF	Acute myocardial infarction	*Depressive disorder*	–
8	45	Male	Blood and PF	Possible cardiac death	*Paranoid Schizophrenia*. Valproic acid	–
9	60	Female	Blood, urine, gastric content, hair, PF and nail	Suicide	Ketazolam and bromazepam	Venlafaxine [0.28]
10	67	Male	Blood, urine and PF	Choking suffocation	Olanzapine	Venlafaxine [0.28] and olanzapine <LLOQ
11	37	Male	Blood, urine, bile, vitreous humor and PF	–	*Drinker and smoker*	–
12	32	Male	Blood, urine and PF	Fall	*Depressive disorder*. Haloperidol, olanzapine and flufenazine,	Venlafaxine [0.27] and olanzapine [0.23]
13	64	Female	Blood, urine, hair, vitreous humor, bile and PF	Methanol poisoning	–	–
14	65	Male	Blood, urine, gastric content and PF	Suspected suicide	Heptaminol hidroclorure, sertraline	–
15	83	Male	Blood, urine and PF	Suicide	Pregabaline, carbamazepine, clonazepam and mirtazapine	Mirtazapine [0.19]
16	48	Male	Blood, urine and PF	Adverse drug reaction	Fluoxetine, lormetazepam, lorazepam, alprazolam, clozapine, cocaine and dipotassium chlorazepate	Fluoxetine [0.63]
17	75	Female	Blood, urine, gastric content and PF	Drug overdose	Tramadol, ciclobenzaprine, ketazolam and lorazepam	Venlafaxine [8.48]
18	70	Female	Blood, urine and PF	Suicide	Venlafaxine and lorazepam	Venlafaxine [0.81]
19	89	Female	Blood, gastric content and PF	Previous depressive episodes	Lorazepam and olanzapine	Olanzapine <LLOQ
20	75	Female	Blood, urine and PF	Natural	Alprazolam, trazodone, venlafaxine and quietiapine	Venlafaxine [0.95]
21	47	Female	Blood, urine and PF	Natural	*Depressive and personality disorder*. Alprazolam and paroxetine	–
22	56	Male	Blood, urine and PF	Cardiac death	–	–
23	35	Male	Blood, vitreous humor and PF	Choking suffocation	Benzodiacepines, topiramate, fluoxetine, paliperidone, quetiapine and mirtazapine	Mirtazapine [0.39] and fluoxetine [0.20]
24	36	Male	Blood, urine, vitreous humor and PF	Suicide	*Depressive disorder*	–
25	61	Male	Blood, urine, vitreous humor and PF	Natural	*Bipolar disorder. Alcohol consumption*. Benzodiacepines, pregabaline, sertraline and olanzapine	Sertraline [0.23]
26	86	Male	Blood, urine, vitreous humor and PF	Carbon monoxide poisoning	Sertraline, trazodone and lorazepam	Sertraline [0.48]
27	73	Female	Blood, urine, vitreous humor and PF	Suffocation from airway occlusion	Benzodiacepines and venlafaxine	Venlafaxine [0.62]
28	60	Female	Blood, urine and PF	Empyema	*Alcohol consumption*. Mirtazapine, quetiapine and benzodiapcepines	Citalopram [0.27]
29	40	Female	Blood, vitreous humor and PF	Suicide	*Depressive disorder*. Venlafaxine, benzodiacepines, clomipramine and trazodone,	Venlafaxine [3.84]
30	55	Female	Blood, vitreous humor and PF	Natural	–	–
31	52	Male	Blood, urine, vitreous humor and PF	Suicide	*Depressive disorder*	Venlafaxine [0.33]

PF, pericardial fluid.

## Discussion

The present work aims to contribute toward the development of methods able to quantify the presence of antidepressants in samples of PF in cases where adequate blood samples may not be available. Previous literature reports comprise only a few articles where PF is used as a biological sample for the determination of drugs (7, 8, 15). The DLLME was one of the techniques used by several authors to extract antidepressants, but only one author determined the concentration of these compounds in PF (8, 16, 20–30). Most of the articles published so far used the DLLME technique coupled to another extraction method (16, 20, 22–24, 26, 27), but few papers have followed a similar method to ours (21, 25, 28–30). Most of them employed urine as the biological matrix (25, 28–30), while Lima et al. used water samples (21). Most of the published research dealt with TCAs as the particular drugs under study (16, 20, 25–27, 29, 30), and only two articles have employed GC–MS (22, 29).

On the grounds of the available evidence, it can be stated that drug concentrations in PF are mostly equivalent to those in peripheral blood (7–9, 31). In consequence, samples of PF can be considered as an alternative for drug quantification in cases where blood samples are not available. PF is usually available in quantities large enough, thus making it suitable for toxicological purposes. In addition, rather similar treatments and procedures to those customarily used for blood can be followed, a fact that constitutes an added advantage for the use of this particular fluid. Finally, as the PF is found within a closed compartment, contamination by microorganisms is less likely to occur (8) than in case of other samples.

A set of experiments were carried out in the quest for an efficient antidepressant extraction procedure by varying several DLLME parameters as the quantity of water, salt amounts, types of extraction and disperser solvents and quantity of biological matrix used. With respect to the volume of sample used, our method uses 0.3 mL, while other authors employ larger volumes (7, 9, 31). The use of chloroform as extracting solvent and acetonitrile as disperser solvent was also determined by Fernández et al. in a study published in 2016 (23). Finally, the method here developed was fully validated with good results in accordance with the limits approved by the FDA. The developed method was used to determine 31 PF samples. The results confirmed the presence of venlafaxine in most cases (12 cases), followed by olanzapine (4 cases), mirtazapine (3 cases), fluoxetine and sertraline (2 cases) and citalopram (1 case). No positive cases for paroxetine have been found. Fernández et al. also found venlafaxine as the most frequently used antidepressant in his study (23). However, citalopram and mirtazapine were the two antidepressants most frequently detected in Leere et al.’s study (8) followed by venlafaxine and sertraline. In the study just referred to, venlafaxine was detected in six cases, having found within four of those cases drug concentrations above the therapeutic range. In addition, one of the cases here studied revealed the presence of extremely high concentrations. Paroxetine was also poorly detected (8). In our study, high concentrations of venlafaxine were also found in three cases. All antidepressants, except one, were found in concentrations above LLOQ. Of the four cases detected for olanzapine, three of them were detected at concentrations below LLOQ.

In view of the present results, we recommend that PF should be added to the list of biological samples used in a forensic laboratory. To conclude, it is worth remarking the increase in use of PF in toxicological practice as pointed out by Contreras et al. in their analysis of morphine and cocaine (11, 32).

## Conclusions

The aim of the present article is to report on an optimized analytical method to determine antidepressants using PF as a biological matrix. Sample preparation based on DLLME was employed. An experimental design setup using StatGraphics 18 and 175 µL of chloroform and 750 µL of acetonitrile as the best quantities for the extracting and disperser solvents was employed. A gas chromatograph using HP5-MS capillary column was used for the separation, and a mass spectrometer was used for the identification. Analytes were identified by their retention times and mass spectra. The method provides high precision, accuracy and recovery within the linear range of detection. Therefore, our method was found to be specific, sensitive and selective enough for the routine analysis of antidepressants in PF and for the application of the method in forensic practice. In conclusion, we have shown the usefulness of PF samples in cases where adequate blood specimens cannot be made available. The existing literature regarding drug concentrations in PF is however scarce, so further studies are in order.

## Supplementary Material

bkab003_Supp
