# Supplementary material for: Determination of Seven Antidepressants in Pericardial Fluid by Means of Dispersive Liquid–Liquid Microextraction and Gas Chromatography–Mass Spectrometry
Source: J Anal Toxicol. 2021 Jan 7;46(2):146–56. doi: 10.1093/jat/bkab003 (PMC8866815; doi:10.1093/jat/bkab003)
Supplement: bkab003_Supp [file bkab003_supp.zip › jat-20-3280-File002.doc]

|  | **Intra-day study (n=5)** | | **Inter-day study (n=5)** | |
| --- | --- | --- | --- | --- |
| **Concentrations (μg/mL)** | **Mean relative error (%)** | **RSD (%)** | **Mean relative error (%)** | **RSD (%)** |
| **FLUOXETINE** | | | | |
| 0.2 | 17 | 5 | 14 | 10 |
| 5 | 6 | 5 | 8 | 4 |
| 10 | 6 | 7 | 1 | 5 |
| **VENLAFAXINE** | | | | |
| 0.02 | 18 | 8 | 14 | 7 |
| 5 | 6 | 3 | 5 | 3 |
| 10 | 2 | 5 | 1 | 4 |
| **MIRTAZAPINE** | | | | |
| 0.02 | 19 | 3 | 16 | 6 |
| 2.5 | 11 | 2 | 6 | 2 |
| 5 | 1 | 4 | 3 | 6 |
| **SERTRALINE** | | | | |
| 0.2 | 17 | 7 | 18 | 7 |
| 2.5 | 8 | 5 | 6 | 3 |
| 5 | 2 | 8 | 1 | 4 |
| **CITALOPRAM** | | | | |
| 0.04 | 17 | 10 | 13 | 7 |
| 2.5 | 5 | 4 | 4 | 3 |
| 5 | 1 | 4 | 2 | 3 |
| **PAROXETINE** | | | | |
| 1 | 13 | 12 | 13 | 12 |
| 5 | 8 | 15 | 9 | 12 |
| 10 | 14 | 12 | 1 | 7 |
| **OLANZAPINE** | | | | |
| 0.2 | 12 | 14 | 19 | 5 |
| 5 | 14 | 3 | 9 | 6 |
| 10 | 6 | 3 | 2 | 7 |

Table 3. Intra- and inter-day precision and accuracy (mean relative error) of the method.

|  | **Intra-day study (n=5)** | **Inter-day study (n=5)** |  |
| --- | --- | --- | --- |
| **Concentrations (μg/mL)** | **Recovery (%)** | **Recovery (%)** |  |
| **FLUOXETINE** | | | |
| 0.2 | 98 | 89 |  |
| 5 | 93 | 91 |  |
| 10 | 94 | 100 |  |
| **VENLAFAXINE** | | | |
| 0.02 | 92 | 95 |  |
| 5 | 93 | 95 |  |
| 10 | 98 | 99 |  |
| **MIRTAZAPINE** | | | |
| 0.02 | 85 | 94 |  |
| 2.5 | 89 | 94 |  |
| 5 | 92 | 97 |  |
| **SERTRALINE** | | | |
| 0.2 | 97 | 98 |  |
| 2.5 | 91 | 94 |  |
| 5 | 98 | 99 |  |
| **CITALOPRAM** | | | |
| 0.04 | 93 | 105 |  |
| 2.5 | 95 | 96 |  |
| 5 | 99 | 98 |  |
| **PAROXETINE** | | | |
| 1 | 93 | 93 |  |
| 5 | 91 | 91 |  |
| 10 | 94 | 101 |  |
| **OLANZAPINE** | | | |
| 0.2 | 89 | 89 |  |
| 5 | 85 | 90 |  |
| 10 | 87 | 98 |  |

Table 4. Intra and inter-day accuracy (analytical recovery) of the method.
